# Establishing a High-Quality Pediatric Cardiac Surgery Program in Post-Conflict Regions: A Model for Limited Resource Countries

**DOI:** 10.1007/s00246-023-03384-7

**Published:** 2024-01-19

**Authors:** Tammam Youssef, Fouad Bitar, Hassanain Alogla, Maya El Khoury, Jihan Moukhaiber, Farah Alamin, Bassam AlHareth, Cristoveanu Catalin Gabriel, Rana Youssef, Labib Abouzahr, Zahi Abdul Sater, Fadi Bitar

**Affiliations:** 1 San Donato Milanese-Milano, Italy; 2 Children’s Heart Center, Department of Pediatrics and Adolescent Medicine, American University of Beirut, Beirut, Lebanon; 3 Cardiac Surgery Program at Imam Al Hassan Hospital, Karbala, Iraq; 4 Marie Curie Children’s Hospital Bucharest, Bucharest, Romania; 5 University Tichreen Hospital, Latakia, Syria; 6 Labib Medical Center, Sidon, Lebanon; 7 College of Public Health, Phoenicia University, Mazraat El Daoudiyeh, Lebanon; 8 Beirut Global Foundation for Congenital Heart Disease, Beirut, Lebanon

**Keywords:** Congenital heart surgery, Limited resource country, Building in situ programs, Outcome of pediatric cardiac surgery, Governmental and private partnership, low- and middle-income countries

## Abstract

**Background:**

Congenital Heart Disease stands as a prominent cause of infant mortality, with notable disparities in surgical outcomes evident between high-income and low- to middle-income countries.

**Objective:**

This study presents a collaborative partnership between a local governmental entity and an international private organization to establish a high-quality Pediatric Cardiac Surgery Program in a post-conflict limited resource country, Iraq.

**Methods:**

A descriptive retrospective study analyzed pediatric cardiac surgery procedures performed by a visiting pediatric heart surgery team from October 2021 to October 2022, funded by the Ministry of Health (MOH). We used the STS-EACTS complexity scoring model (STAT) to assess mortality risks associated with surgical procedures.

**Results:**

A total of 144 patients underwent 148 procedures. Infants comprised 58.3% of the patients. The most common anomalies included tetralogy of Fallot, ventricular septal defect, and various single ventricle categories, constituting 76% of the patient cohort. The overall surgical mortality rate was 4.1%, with an observed/expected surgical mortality rate of 1.1 (95% CI 0.5, 2.3). There was no significant difference between our observed surgical mortality in Category 2, 3, and 4 and those expected/reported by the STS-EACTS Database (*p* = 0.07, *p* = 0.72, and *p* = 0.12, respectively). The expenses incurred by the MOH for conducting surgeries in Iraq were lower than the alternative of sending patients abroad for the same procedures.

**Conclusion:**

The partnership model between a local public entity committed to infrastructure development and funding and an international private organization delivering clinical and training services can provide the foundation for building sustainable, high-quality in situ programs in upper-middle-income countries.

## Introduction

Congenital Heart Disease (CHD) significantly contributes to infant mortality, with a global incidence rate of approximately 10 per 1,000 live births [1, 2]. CHD imposes a heavy burden, particularly in regions with higher fertility rates and lower per capita income, where economically disadvantaged areas account for 90% of global CHD cases [2]. Each year, approximately 1.5 million infants are born with CHD worldwide [2–5], and their chances of survival are influenced by geographical location and access to medical resources. Regrettably, 90% of those born with CHD in low- and middle-income countries (LMICs) lack access to essential cardiac care, resulting in higher mortality and disability rates compared to high-income nations [1, 5–7].

The inadequacy of pediatric cardiac surgery services is a prevalent issue in most LMICs, exacerbating disparities in CHD outcomes between high-income countries (HICs) and LMICs. The healthcare landscape in low- and middle-income nations faces numerous challenges, including limited access to advanced facilities, a shortage of skilled healthcare professionals, inadequate infrastructure, financial constraints, and governance limitations [8–11]. These challenges hinder the provision of adequate pediatric cardiac care in LMICs. Financial affordability poses a significant barrier to accessing care, especially for high-cost interventions like congenital heart surgery.

Various initiatives and partnerships have been established to address disparities in pediatric cardiac care, aiming to enhance access to essential care and reduce CHD-related mortality in LMICs. Collaboration and coordination among pediatric cardiac non-governmental organizations (NGOs) have been crucial in addressing healthcare barriers in LMICs. These NGOs work towards strengthening local clinical capacity, providing technical assistance, and advocating for improved outcomes data to establish the legitimacy of their efforts with policymakers and donors [3–5, 7, 10, 12–24].

Iraq, an upper-middle-income, post-conflict nation, allocates 4.2% of its Gross Domestic Product (GDP) to healthcare [25]. Few hospitals in Iraq conduct pediatric cardiac surgery, and most are performed by visiting pediatric cardiac surgeons. Local Iraqi pediatric cardiac surgeons are limited, primarily handling simple and moderately complex cases. Complex and infant pediatric cardiac surgeries are performed by visiting teams or referred for surgeries abroad. The country has recently made strides in healthcare infrastructure. Selective governmental and private hospitals in Iraq now have the infrastructure to perform pediatric open-heart surgeries. Building on this progress, we have launched an international organization that addresses CHD care in countries with limited resources like Iraq. Our mission focuses on delivering clinical and educational pediatric cardiology and CHD services. We have successfully established a high-quality Pediatric Cardiac Surgical Program with Iraq’s Ministry of Health.

This report will provide an in-depth analysis of our initiative’s experience, outcomes, and challenges. By doing so, we hope to offer a potential roadmap for the surgical management of CHD in LMICs, demonstrating how collaborative efforts can overcome the myriad of challenges healthcare systems face in these regions.

## Methods

### Study Design and Setting

This retrospective cohort study was conducted at two hospitals in Karbala, Iraq: a newly established governmental hospital and a private, not-for-profit hospital contracted by the Ministry of Health (MOH). The surgeries under investigation were performed between October 2021 and October 2022 and were sponsored by the Ministry of Health in Iraq. Our international visiting team comprises members from multiple countries (Lebanon, Italy, Romania, Syria, and France). The team operates under the umbrella of a Lebanese foundation mandated to provide clinical, educational, and training services.

### Patient Selection

Before the surgeries, our team conducted a comprehensive assessment of the hospital facilities to ensure that safe surgical procedures could be carried out, particularly for the governmental hospital, which was initiating its pediatric cardiac surgery program. The MOH provided a list of patients referred from various regions in Iraq, with their medical summaries and echocardiography reports. In previous years, the Ministry of Health used to send these patients abroad for surgical intervention. Upon evaluation, our visiting team identified the patients who would undergo surgery during the visit period. We excluded newborns weighing less than 2.5 kg and patients requiring Fontan surgeries. Fontan patients were excluded due to logistical constraints from our visiting team’s stays, typically lasting a couple of weeks. Given the potential for extended hospitalization in Fontan cases, we strategically deferred these procedures to ensure continuous and comprehensive care.

### Surgical Facilities

Surgeries were performed at two distinct centers: private, not-for-profit, and newly established governmental hospitals. Both facilities possessed the necessary infrastructure and medical equipment for pediatric open-heart surgeries. Our visiting team supplied the consumables required for the surgeries.

### Data Collection and Analysis

Data were extracted from patient medical records and included demographics, surgical procedures, complexity class, mortality rates, and infection rates. Patients were categorized based on their cardiac defects and the surgical procedures they underwent. Patients were labeled according to the highest complexity class in multiple defects or repair cases.

We employed the STAT Categories, ranging from 1 to 5, to classify the surgeries based on their complexity, with STAT-1 being the least complex and STAT-5 the most. These categories were developed using data from The Society of Thoracic Surgeons (STS) and The European Association for Cardio-Thoracic Surgery (EACTS). The STS-EACTS complexity scoring model (STAT) was used to assess mortality risks associated with surgical procedures [26].

In-hospital surgical mortality was categorized by procedure and risk levels. These outcomes were then compared to the benchmark mortality risks outlined by the STS-EACTS model using the STAT Mortality Score/Categories [26]. All outcome measures and surgical morbidity and mortality rates were audited and validated by a designated committee at the MOH in Iraq and the local hospital team.

The study received ethical approval from the cardiac surgical department and the Ministry of Health (MOH) committees overseeing the project. Ethical guidelines and regulations were conducted in all study aspects to ensure patient confidentiality and data integrity.

### Training and Capacity Building

After our initial visit, our team seamlessly integrated with local professionals, such as nurses, physicians, and perfusionists. We introduced a structured framework to enhance the local team’s capabilities, emphasizing a gradual and collaborative approach to skill development. Our action plan included assessing their skills, evaluating resources, and identifying areas for improvement. The hospital staff comprises local nurses, a perfusionist with adult experience, and surgeons with limited exposure to pediatric cardiac surgery. With the Ministry of Health intending to designate the governmental hospital as a training center, we developed a comprehensive curriculum, progressively increasing responsibilities through workshops and shadowing. Local ICU nurses, operating room staff, and the cardiac surgeon advanced in their roles, and future plans involve training anesthesiologists and cardiac intensivists. Our team fostered a culture of teamwork, safety, and the establishment of a robust system.

### Statistical Analysis

Data analysis utilized Microsoft Excel 2016^©^. To assess statistical significance in mortality rates between the two groups, either the Chi-square or Fisher’s exact test was employed at a specified confidence level, such as *p* < 0.05, with a 95% confidence interval.

## Results

### Demographics and Patient Distribution

Six visits were conducted during the one-year study period, each lasting approximately two weeks. A total of 144 patients were treated, undergoing 148 surgical procedures, all performed by a single surgeon. The patient population exhibited a near-equal gender distribution, with 49% girls and 51% boys. Most patients (58.3%) were infants, with 34% being under six months of age and 22% weighing less than 5 kg. (Table 1).

**Table 1:** Patients’ age, weight, and sex distribution

Variables	No. (%)
Gender	
Grils	73 (49%)
Boys	71 (51%)
Age	
< 6 months	49 (34.0%)
6–12 months	35 (24.3%)
> 1 year	60 (41.7%)
Weight	
Less than 5 kg	31 (21.5%)
5–9 kg	67 (46.5%)
> 9 kg	46 (31.9)

### Types of Cardiac Anomalies and Procedures

**Fig. 1:**
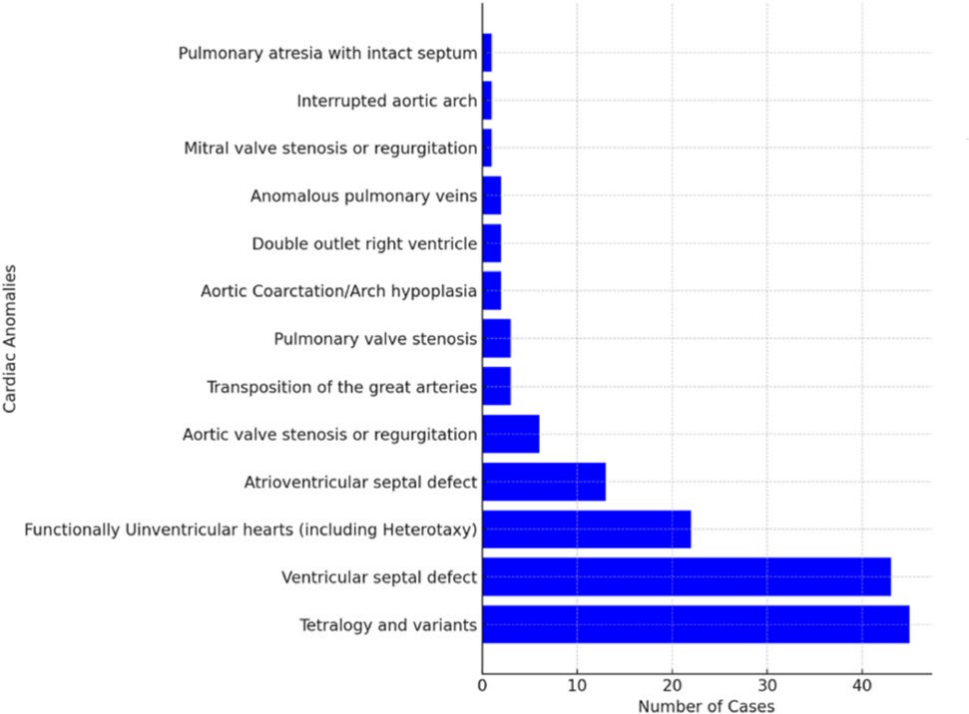
List and distribution of cardiac anomalies

The most common anomalies included tetralogy of Fallot, ventricular septal defect, and various single ventricle categories, constituting 76% of the patient cohort (Fig. 1).

**Fig. 2:**
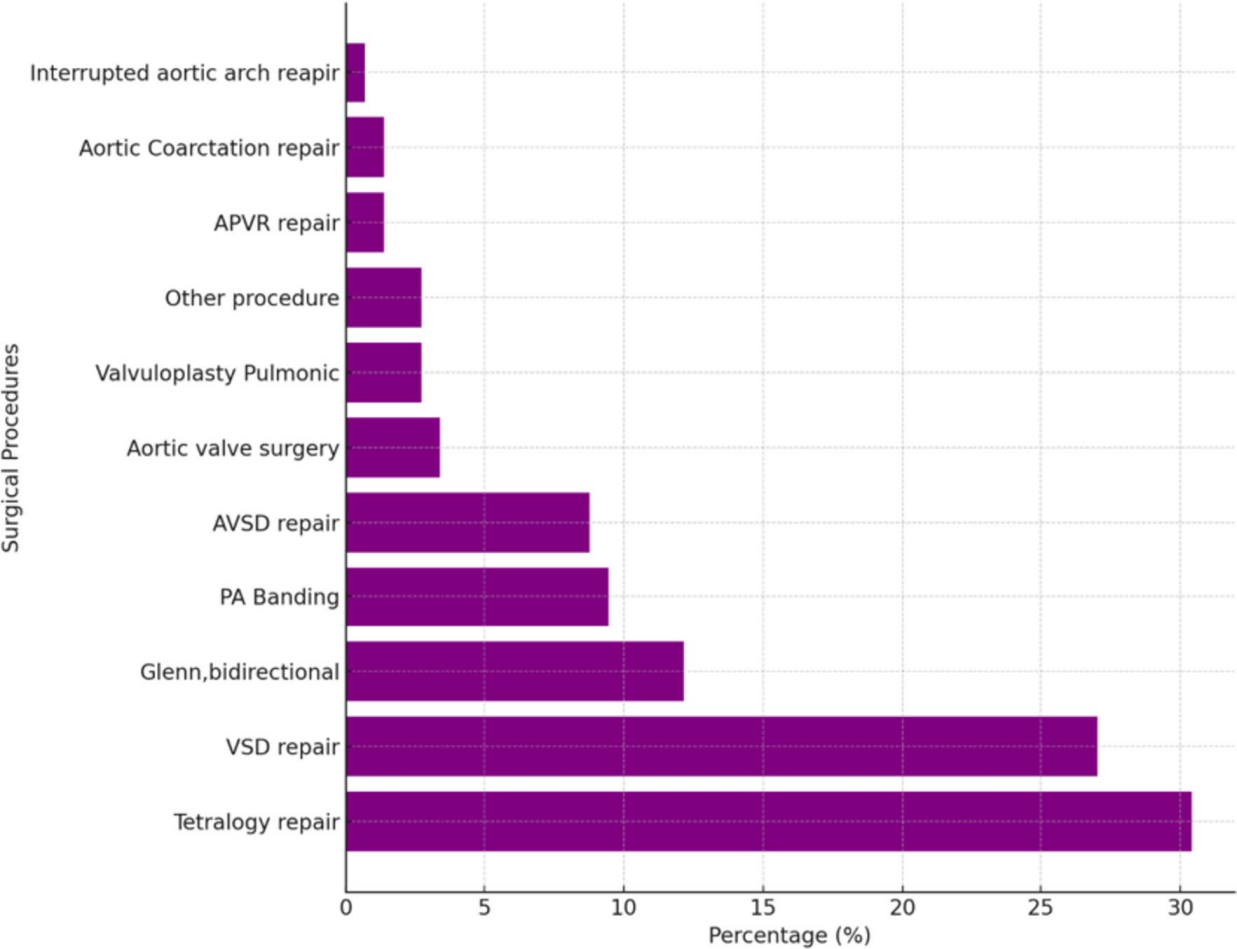
List of surgical procedures

Among the 148 procedures, TOF repair was the most common (30.4%), followed by VSD closure (27%), Bidirectional Glenn (12.2%), and pulmonary artery banding (PAB) at 9.5% (Fig. 2). Our TOF repair policy prioritized valve-sparing when feasible. The distribution of these procedures across various complexity categories is detailed in Table 2 and compared with STAT data.

**Table 2:** Operative mortality. Comparison of International Visiting Pediatric Cardiac Surgery Team (IVT) vs. Combined EACTS data and STS data

STS-EACTS category	Death No./number of operated patients	Observed Morality (IVT)	Expected morality combined STS-EACTS	Observed/Expected ratio	*P* value 95% CI
1	1/43	2.3%	0.78%	2.9	0.03
2	2/73	2.7%	2.1%	1.3	0.07
3	1/15	6.6%	3.4%	1.9	0.72
4	2/17	11.8%	8.5%	1.4	0.12
5	–	–	19.9%	–	
Overall	6/148	4.1%	3.8%	1.1 (0.5; 2.3)	0.54

### Complications and Mortality

**Fig. 3:**
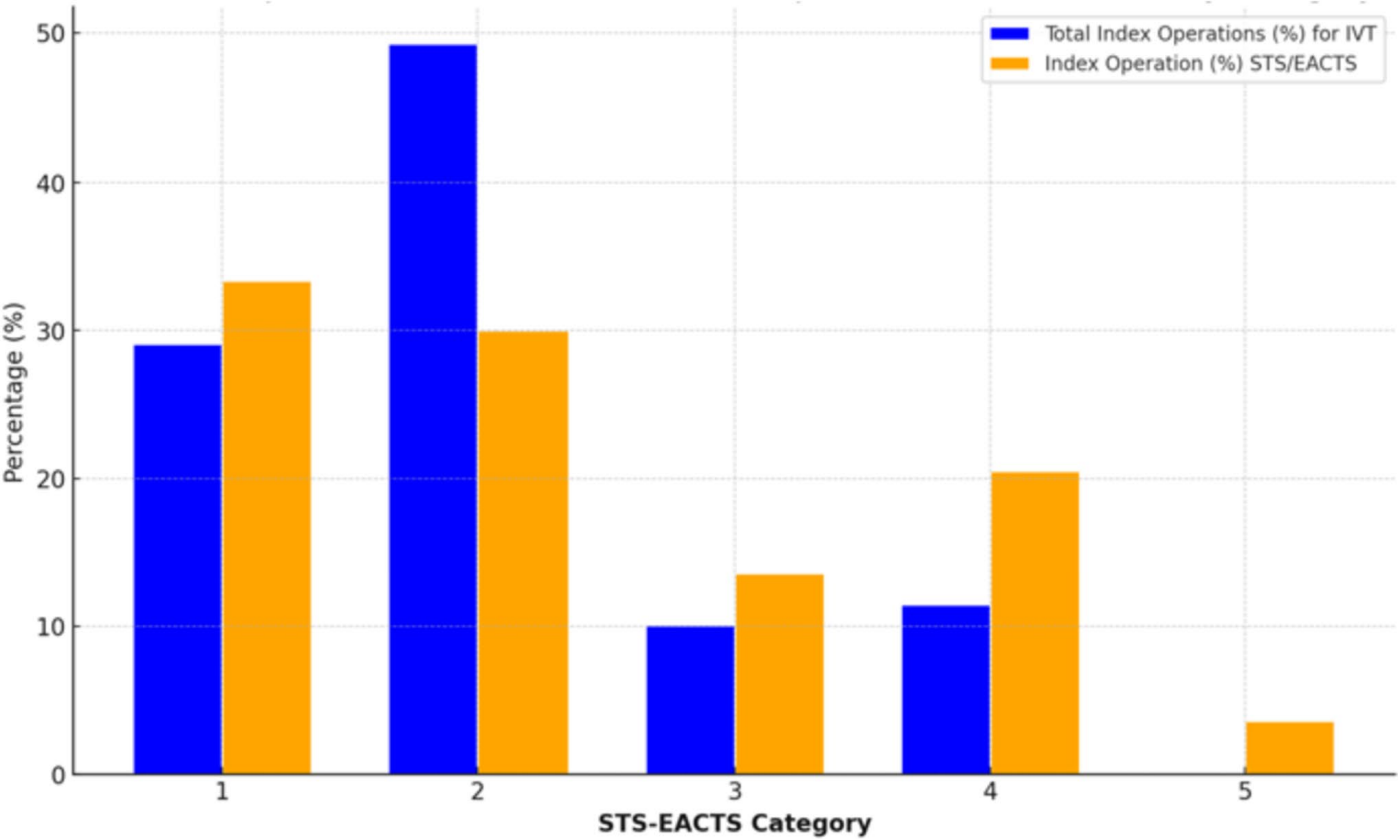
Comparison of the distribution of the indexed operation (%) per STAT categories between the surgeries performed by the International Visiting Team (IVT) and the index operation (%) reported by the SET/EACTS data

The average length of ICU stay was 5.7 days (± 6.2 days), and the total hospital stay averaged 12.2 days (± 8.8 days). Mortality rates were assessed based on the STAT categories, ranging from 1 (lowest risk) to 5 (highest risk). In our cohort, 23% fell into the complex STAT categories 3 & 4, while 49% were in Category 2 (Fig. 3). The overall discharge surgical mortality rate was 4.1%, with an observed-to-expected mortality ratio of 1.1 (95% CI 0.5; 2.3). No significant difference was found between our surgical mortality rates and those reported in the STS–EACTS Database [26].

Our overall discharge surgical mortality was 4.1%. Our observed/expected surgical mortality rate was 1.1, with a 95% confidence interval of (0.5;2.3). There was no significant difference between our surgical mortality in Category 2, 3 and 4 and those reported by the STS–EACTS Database (*p* = 0.07, p = 0.72 and *p* = 0.12, respectively) [26]. One out of 43 patients died in Category 1 with a *p* = 0.03). Our surgical mortality rate is not statistically different from the STS mean score with a p-value of 0.54. (Table 2).

### Causes of the In-hospital Mortality

Six patients succumbed post-surgery. Three deaths were attributed to infections, two occurring more than four weeks post-surgery. Specific cases included an infant with Pulmonary Atresia/VSD, a 4-month-old with SV, S/P BDG, and a third baby with S/P PAB. Additional mortalities involved complex CHD cases, including one with an Interrupted Aortic Arch and TGA + VSD (STAT 4 Category) and another with a complex Univentricular Heart (STAT 4 Category). One patient with AVC died due to a pacemaker-related issue.

### Cost Analysis

The Ministry of Health (MOH) allocated a lump sum ranging from 8,600 to 9,000 USD per case, covering facilities, infrastructure, and medical equipment. This comprehensive cost from the MOH encompasses various elements, including travel expenses, accommodation, food, surgical consumables, and fees for the visiting multidisciplinary team. The team, consisting of surgeons, cardiologists, intensivists, nurses, anesthesiologists, and perfusionists, contributed to the collaborative effort. Given that the program will conduct more complex neonatal surgeries, the Ministry of Health recognizes that the current lump sum cannot cover all costs associated with these complex cases. There is a plan to increase the coverage to an adequate amount, enabling the performance of these surgeries within Iraq.

## Discussion

Many challenges undeniably amplify the burden of Congenital Heart Disease (CHD) in LMICs. These challenges encompass limited resources, governance inefficiencies, and a lack of access to specialized medical care [2, 9, 27, 28]. While various models have been devised to address CHD, such as overseas treatment [29–32], humanitarian missions [25, 33, 34], and NGO support, they frequently grapple with sustainability issues, hindering their long-term effectiveness [34].

Our “Beirut Global Foundation for Congenital Heart Disease” initiative represents a determined effort to confront these challenges head-on by providing comprehensive clinical, educational, and training services. One key aspect of our approach has been collaborating with Iraq’s Ministry of Health to establish a high-quality CHD surgical program, specifically emphasizing surgical interventions and capacity building for local medical teams. This collaborative model leverages the strengths of both local governmental bodies and international organizations, creating a sustainable healthcare ecosystem. Our approach aligns with the “in-situ hybrid model,” which has shown promise in other low- and middle-income countries (LMICs) [21, 23, 35, 36].

Notably, our in-hospital surgical mortality rate of 4.1% aligns closely with international standards, as evidenced by the STS-EACTS Database. Particularly noteworthy is our surgical mortality rate in STAT Categories 2, 3, and 4, which closely resembles those reported by the STS–EACTS Database. Our patient population primarily consists of individuals with moderate complexity (STAT Categories 3 and 4), including a substantial percentage of infants. This demonstrates the feasibility of delivering high-quality surgical care in resource-limited settings and underscores our mission’s urgency.

Importantly, our dataset contributes to the limited data reported by visiting surgical teams that compare mortality rates to the STS/EACTS Database. This highlights the critical importance of implementing a structured reporting system for international humanitarian missions, NGOs, and organizations to facilitate quality improvement efforts.

Local medical teams in LMICs can effectively handle relatively simple surgical cases with favorable outcomes [35, 37–39]. Similarly, visiting surgical teams have succeeded in low-resource countries, mainly when avoiding neonatal and complex surgeries [12, 15, 24, 40–43]. Our experience supports that favorable surgical outcomes and successful programs are attainable in limited resource countries, even for moderately complex lesions and infants.

A cornerstone of our model is the emphasis on training and education to enhance local healthcare capacity. We aim to cultivate a locally functional, sustainable team capable of independently managing their roles and tasks. Establishing and maintaining an in situ program hinges on cultivating and training sufficient local healthcare professionals to deliver services effectively [4, 12, 44]. This approach is vital for long-term sustainability and addresses the “brain drain,” where skilled professionals migrate to higher-income countries. The public sector must provide competitive compensation to retain these essential medical staff.

Challenges during the program’s initial phase, such as ensuring consumables and infrastructure readiness, were resolved through a preliminary check. Addressing stay-over cases required the presence of some team members. Our role involved capacity building for local medical teams, leading to an enhanced local team experience and increased participation in patient care, including stay-over cases, over time.

In terms of cost-effectiveness, our model demonstrated that the expenses incurred by the Ministry of Health for conducting surgeries in Iraq are lower than the alternative of sending patients abroad for the same procedures, typically performed in a neighboring country. This strategic approach not only optimizes financial resources for the MOH but also underscores the efficiency of conducting these medical procedures within the country. [13]. Our adaptable model can be tailored to individual countries’ unique needs and resources. The cooperative model, where the local government provides infrastructure and funding while international organizations initially contribute human resources and assist in local capacity building, has proven effective in establishing sustainable and high-quality pediatric cardiac programs. This model is particularly suitable for upper-middle-income countries to emulate and can be modified for low-income countries by involving additional stakeholders like NGOs and humanitarian organizations.

Our study was limited by the lack of mid and long-term patient follow-up data, hindering a comprehensive understanding of post-treatment progress. Additionally, we couldn’t provide ongoing program sustainability updates. These limitations highlight the need for future research with extended follow-up periods to assess patient outcomes and program effectiveness over time.

## Conclusion

In conclusion, our collaborative model, which unites local governments in the public sector, committed to infrastructure development and funding, with international private organizations dedicated to delivering CHD-related clinical and training services, has demonstrated significant potential in establishing the foundation for a sustainable and high-quality CHD program in Iraq. This adaptable framework can be tailored to other countries’ diverse geopolitical and socioeconomic contexts.

By sharing our experiences and outcomes, we aim to contribute meaningfully to global efforts to enhance CHD care. We underscore the vital importance of collaboration, capacity building, and sustainability in addressing the challenges posed by congenital heart disease. Through collective efforts, we can make substantial strides in improving the lives of individuals affected by CHD in resource-limited regions worldwide.
